# A targeted long-read sequencing approach questions the association of OXTR methylation with high-functioning autism

**DOI:** 10.1186/s13148-023-01616-4

**Published:** 2023-12-20

**Authors:** Jelte Wieting, Kirsten Jahn, Stefan Bleich, Helge Frieling, Maximilian Deest

**Affiliations:** 1 Hannover Medical School, Department of Psychiatry, Social Psychiatry and Psychotherapy, Carl-Neuberg-Str. 1, 30625 Hannover, Germany; 2 Laboratory for Molecular Neuroscience, Feodor-Lynen-Str. 35, 30625 Hannover, Germany

**Keywords:** High-functioning autism, Oxytocin receptor, OXTR, Nanopore Cas9-targeted sequencing, Long-read sequencing, Methylation, 5mC modification

## Abstract

**Background:**

DNA sequence variation and altered epigenetic regulation of the oxytocin receptor gene (OXTR) have been implicated in autism and autistic-like behaviors. While previous studies have examined subsegments of OXTR, nanopore Cas9-targeted sequencing (nCATS) allows deep characterization of entire genes with simultaneous assessment of epigenetic 5-methylcytosine (5mC) modification and without the need for prior DNA amplification or bisulfite conversion. This pilot study uses an nCATS approach to sequence the entire OXTR gene and its regulatory construct and screen for 5mC modification to compare results between individuals with high-functioning autism (HFA) and neurotypical controls (NC).

**Methods:**

Using DNA extracted from peripheral blood, OXTR (Hg38, chr3: 8750381–8770434, 20,054 base pairs) was analyzed by nCATS. 5mC modification probabilities were calculated and visualized across the gene and differential methylation analysis was performed.

**Results:**

Twenty adults with HFA (10 males, 10 females) and 20 age- and sex-matched NC (± 5 years) were included. There were no apparent group differences in the entire OXTR gene sequence, except for the intron variant rs918316, which was clustered in the HFA group. However, differential methylation analysis did not reveal a single significant group-dependent differentially methylated site among the 412 CpG sites captured.

**Limitations:**

Limitations of this study include the small number of samples due to the pilot nature of the study, which particularly limits the relevance of the sequence variants found. It should also be noted that the use of peripheral blood material limits the ability to draw conclusions about central processes.

**Conclusions:**

Previous findings of autism-associated OXTR epigenetic alterations were not reproducible with our method. In our opinion, this may lead to a reconsideration of the relevance of altered methylation at individual OXTR CpG positions in autism research. However, given the pilot nature of the study, these results need to be replicated in independent cohorts and with larger sample sizes.

**Supplementary Information:**

The online version contains supplementary material available at 10.1186/s13148-023-01616-4.

## Background

Among other functions, the neuropeptide oxytocin plays an important role in social bonding, which is impaired in individuals with autism spectrum disorder (ASD), a neurodevelopmental disorder characterized by deficits in social interaction and communication. Preliminary studies suggest that changes in blood oxytocin expression in autistic [1] but also neurotypical individuals predict social deficits [2]. Therefore, genes related to the oxytocin system, in particular the oxytocin receptor gene OXTR, are among the most studied genes in autism research.

There are a number of previous publications, which have looked at the association of changes in OXTR with ASD, including in high-functioning autism (HFA) such as the former Asperger syndrome. Meta-analyses have shown significant effects of several single-nucleotide polymorphisms (SNPs) within the OXTR sequence on ASD [3], some of which, e.g., SNP rs53576, an A to G polymorphism within OXTR intron 3, have also been associated with altered epigenetic regulation of the OXTR gene [4].

This involved one of the most important epigenetic DNA regulatory mechanisms, cytosine methylation, in which a methyl group is added to the carbon 5 position in the cytosine ring (5mC) in the context of so-called CpG dinucleotides (i.e., a cytosine followed in the base sequence by a guanine linked by a phosphate). Commonly found in the region around the transcription start site, CpG-rich gene regions, also known as CpG islands, are thought to have an important gene regulatory function, as higher DNA methylation is generally associated with reduced gene transcription and consequently lower expression [5].

Altered 5mC modification was frequently found in individuals with ASD and was also associated with altered autistic-like behavior [6]. Differential methylation of OXTR in ASD has been found across different age spectrums. Some studies suggest a sex-dependent effect [7, 8]. While most of the previous studies used DNA from peripheral blood material, differential OXTR methylation associated with autism has been demonstrated in various tissues—in saliva [9], but also in postmortem brain tissue [10]. In particular, differentially methylated CpGs in the so-called OXTR MT2 gene region have been repeatedly linked to autism and autism typical behaviors. The 405 bp MT2 region, first described by Kusui et al., is located within the OXTR CpG island and the OXTR promoter region, overlapping exon 1 and intron 1 and containing 26 CpG sites [11]. The methylation levels of these sites have been shown to be closely correlated with the regulation of OXTR expression [12, 13]. To a lesser extent, studies have focused on DNA methylation in a region of exon 3, which has also shown associations with autistic-like behavior [4, 14], although DNA methylation of exon 3 is also thought to depend on methylation of the MT2 region [13].

However, it should be noted that previous studies were based on short-read sequencing methods, which allow sequencing and methylation analysis of small gene fragments and thus do not provide a comprehensive overview. In addition, the commonly used bisulfite sequencing technique only allows indirect determination of methylation only after prior chemical base conversion and subsequent amplification. Third-generation targeted long-read sequencing technologies, such as nanopore Cas9-targeted sequencing (nCATS), allow the characterization of entire genes, including their regulatory architecture, by the simultaneous assessment of single-nucleotide variants, structural variations and CpG methylation [15], without the need for prior PCR amplification or DNA bisulfite conversion, counteracting any information loss due to amplification and base conversion processes.

The present study represents the first attempt to use a targeted nanopore long-read sequencing approach to analyze the sequence and 5mC modification of the entire OXTR gene, including its regulatory elements, and in a second step to compare the results between adults with HFA and neurotypical controls (NC). As sex is known to influence the phenotype of HFA [16] and has also been described to influence 5mC modification of OXTR in autism spectrum disorders [7, 8], we also looked for effects of sex on OXTR 5mC levels.

## Results

### Group characteristics

The study included 20 adults with HFA (10 males, 10 females) and 20 NC (10 males, 10 females). The mean age was 30.5 ± 7.8 y in HFA and 30.7 ± 7.8 y in NC, with an insignificant group difference at p = 0.920. The maximum age was 51 years in HFA and 48 years in NC, while the minimum age was 20 years in HFA and 22 years in NC. There was little group difference in total IQ score, p = 0.080, with 105.4 ± 11.2 points in HFA and 111.6 ± 10.4 points in total WAIS-IV score in the NC group. There was a maximum IQ of 125 points in HFA and 132 points in NC, while the minimum IQ was 88 in HFA and 99 in NC. As expected, the groups differed significantly on the autism-specific self-report psychometric tests AQ and EQ, each at p < 0.001. Table 1 provides a summary of demographic and psychometric data comparing HFA and NC.

**Table 1:** shows the demographics of the HFA and NC groups

	Group			
	HFA	NC		
n =	20	20		
sex	10 male (50.0%), 10 female (50.0%)	10 male (50.0%), 10 female (50.0%)		
			Unpaired t test	
	mean ± SD		t(38)	p
age (in y)	30.45 ± 7.837	30.70 ± 7.794	− 0.101	0.920
IQ total (in points)	105.40 ± 11.217	111.55 ± 10.364	− 1.801	0.080
Autism Quotient	37.85 ± 4.440	13.65 ± 7.407	12.532	** < 0.001**
Empathy Quotient	18.00 ± 8.322	46.45 ± 13.044	− 8.223	** < 0.001**

There were no apparent group differences in age (almost balanced, as matched) and total IQ. As expected, the groups differed significantly on the autism typical psychometrics AQ and EQ

### Nanopore Cas9-targeted sequencing

#### Quality control

DNA was extracted from peripheral blood samples. Two fragments (chr3: 8,761,480–8,749,344 and chr3: 8,770,937–8,763,067) were captured by nCATS using a total of four guide RNAs. Sequence and position of the guide RNAs as well as the corresponding on- and off-target scores can be found in Additional file 1.

The fragment chr3: 8,761,480–8,749,344 showed a lower mean coverage of 20.167 ± 20.092 than the fragment chr3: 8,770,937—8,763,067 (M = 34.221 ± 26.820), which also contains the known gene regulatory units (e.g., MT2 region). The mean coverage of the captured OXTR sequence (including its regulatory built; chr3: 8,750,381—8,770,434) across all samples and positions was 29.233 ± 24.124.

**Fig. 1:**
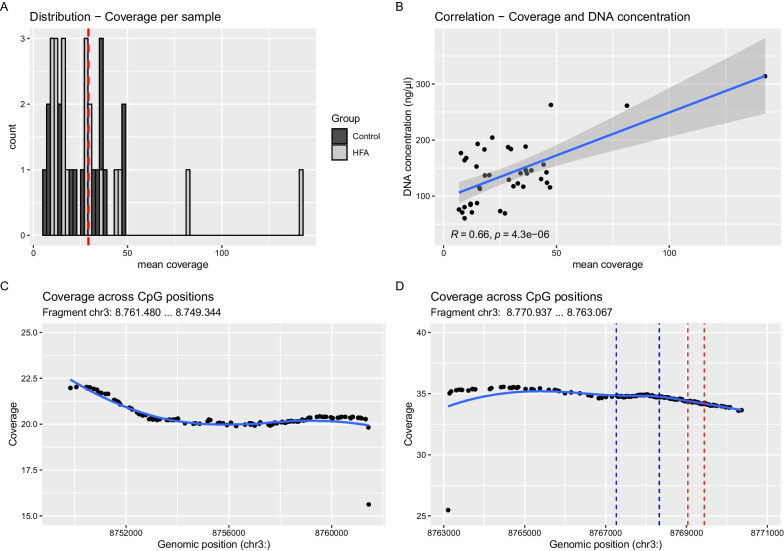
**A** illustrates the distribution of mean coverage per sample within our data, color-coded by group (NC in dark gray, HFA in light gray).** B** shows the correlation between mean coverage and DNA concentration across samples, indicating a positive association. **C** and **D** give the coverage across the genomic region (with CpG positions indicated by black dots) of the guide RNA derived fragments chr3: 8,761,480—8,749,344 (**C**) and chr3: 8,770,937–8,763,067 (**D**). In **D**, the regulatory relevant genomic regions (also displaying a high CpG density) are indicated by dashed lines (blue–Exon 3, red–MT2 area)

The variability in coverage between samples is evident from the distribution plot (Fig. 1A). In the HFA group, there are two outliers with apparently higher coverage (Max = 142.394), which is also reflected in a higher standard deviation in the HFA group. However, there was no statistically significant group difference in mean coverage between HFA and NC, either overall or for either fragment. The mean coverage per sample correlated positively with the concentration of the input DNA, r(38) = 0.66, p < 0.001 (Fig. 1B). However, there was almost constant coverage across individual CpG positions within each of the two fragments (see Fig. 1C, D). Table 2 summarizes the basic coverage statistics.

**Table 2:** shows the basic coverage statistics for the OXTR sequence and for the guide RNA-dependent fragments

		Coverage				t test (coverage~group)		
	Genomic ranges (chr3:)	M	SD	Min	Max	t	df	p
OXTR overall	8,750,381–8,770,434	29.233	24.124	6.790	142.394	− 0.515	26.489	0.611
***HFA***		31.216	31.371	9.240	142.394			
***NC***		27.250	14.217	6.790	47.521			
Fragment 1	8.761.480–8.749.344	20.167	20.092	3.507	125.959	− 0.826	24.427	0.417
**HFA**		22.802	26.654	3.507	125.959			
**NC**		17.533	10.179	3.610	36.240			
Fragment 2	8.770.937–8.763.067	34.221	26.820	6.908	151.238	− 0.378	27.654	0.709
**HFA**		35.841	34.429	9.820	151.238			
**NC**		32.601	16.900	6.908	60.927			

### Methylation analysis

**Fig. 2:**
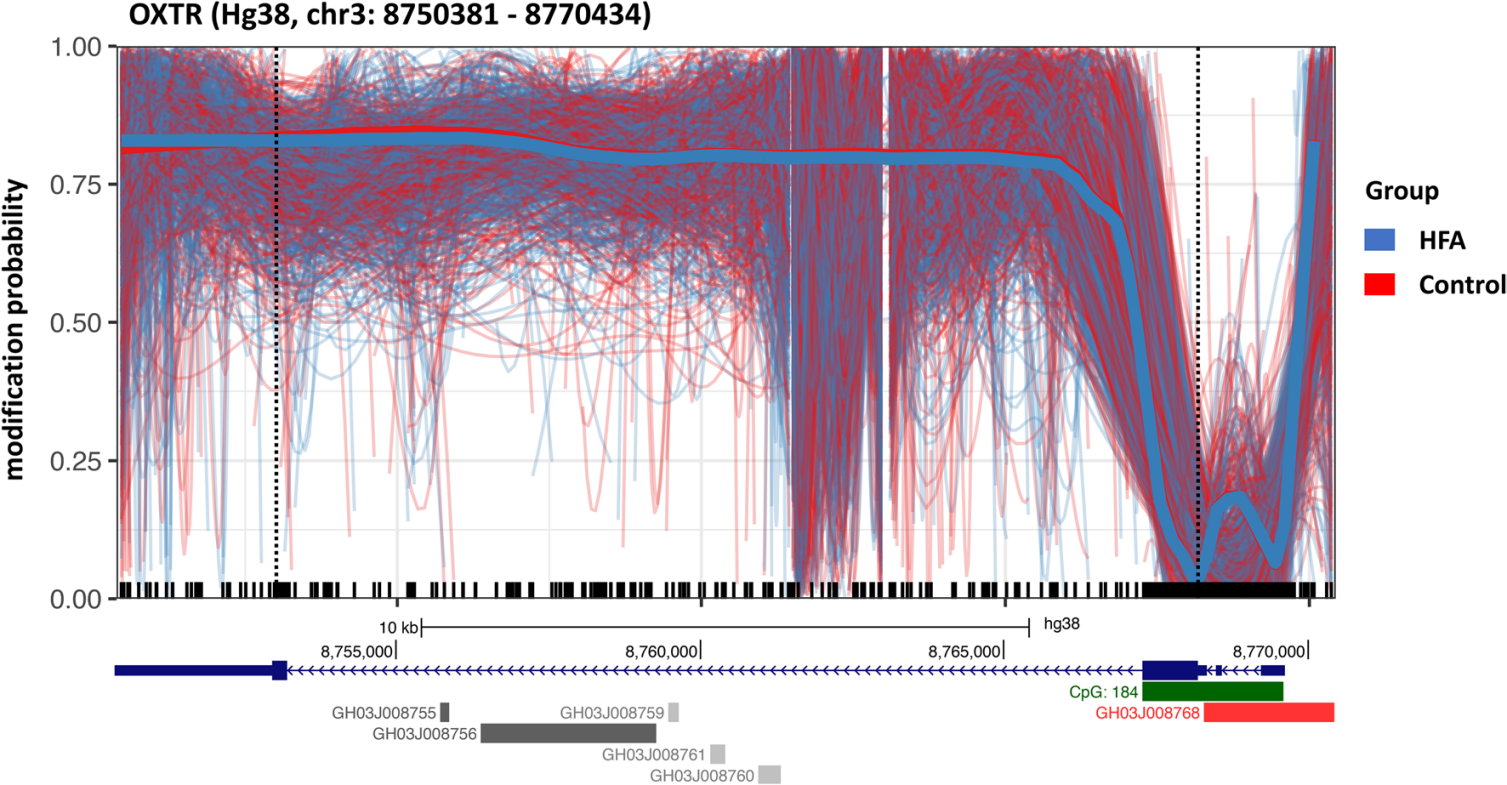
shows the 5mC modification probability across the entire OXTR gene (Hg38:, chr3: 8,750,381–8,770,434, exons represented by dark blue boxes, coding region highlighted in bold in the legend below, transcription start site marked by dotted line) and its regulatory architecture (gene promoter region represented by red box, CpG island represented by green box containing 184 CpG sites and gene enhancer regions represented by gray boxes below). Thin lines in the figure show individual long reads and thick lines show the aggregated trend across all reads, color-coded by group. There were no apparent group differences across the entire OXTR gene, even when focusing on specific regulatory regions

First, the OXTR 5mC modification was examined. We analyzed 412 CpG positions within the OXTR sequence, of which nearly half (n = 184) were located within the 2319 bp OXTR CpG island spanning chr3:8,767,276–8,769,594. Figure 2 shows a spaghetti plot of the entire OXTR gene including all regulatory units. Across the entire OXTR sequence, there were no apparent group differences in methylation. While 5mC modification within the CpG island around the transcription start site and across exons 1–3 appears more dynamic, large portions of the OXTR gene show consistently high methylation across intron 3 and exon 4 in both HFA and NC groups. A region (1.633 bp) within intron 3 (chr3: 8.763.090 to 8.761.458, Hg38) is not covered by the multiple guide approach (indicated by short cuts in the plot), so no valid reads can be expected here.

**Fig. 3:**
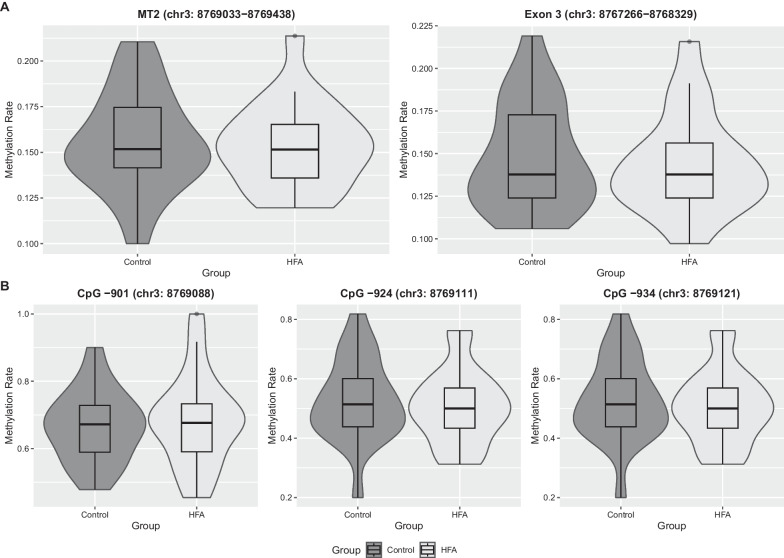
**A** A combined box and violin plot showing the distribution of OXTR MT2 region and exon 3 mean methylation comparing HFA and NC. There is no visually apparent difference in the distribution of the data with an overall statistically nonsignificant difference. **B** Also on a CpG site by CpG site basis, all 26 CpGs examined in the regulatory MT2 region showed no differential methylation. Shown here are the CpGs -901, -924 and -934, which were frequently described as important regulators of OXTR expression

Statistical analysis for differentially methylated regions showed no significant DMRs across the entire OXTR gene including its regulatory relevant units at p < 0.05. Since the CpG island around the transcription start site was found to be more dynamic in methylation, this region was analyzed in detail in a second step. Preliminary studies on methylation of OXTR in ASD based on short reads focused on regions within the CpG island, especially the so-called MT2 region, which has been repeatedly described as regulatory relevant for OXTR [11], and the exon 3 region [6]. There was no significant group difference in 5mC modification across 108 CpGs within OXTR exon 3 with 14.1% ± 2.9% in HFA to 14.8% ± 3.1% 5mC in NC group, p = 0.571. Furthermore, the OXTR regulatory MT2 region (Hg38: chr3: 8,769,033–8769438) showed no significant group difference across 26 CpGs covered with a mean of 15.6% ± 2.4% in HFA and 15.3% ± 2.7% in NC, p = 0.630. Figure 3A shows the distribution of MT2 and exon 3 methylation data as combined box and violin plots.

The analysis for differentially methylated loci also showed no statistically significant hits. We further focused on CpGs previously described to be associated with autism or autism-related traits. According to the given literature, we identified seven CpG sites within MT2 region that have been frequently described in the above-mentioned context (− 860, − 901, − 924, − 934, − 959, − 982, -989 each in positional relation to TSS (transcription start site, Hg38: chr3:8,768,187)). None of these CpG positions differed significantly between the HFA and NC group, with a maximum group difference of 3.7% at CpG -860. In Table 3, based on a recent review on differential methylation of the OXTR gene in ASD (Moerkerke et al., 2021), the differentially methylated CpG positions previously reported in the context of autism were re-investigated in group comparison based on our nanopore long-read sequencing data.

**Table 3:** Analysis of 5mC modification in regions or CpG sites frequently associated with differential methylation in ASD or with autism traits, social cognition alterations or social anxiety (Moerkerke et al., 2021) within our nanopore sequencing dataset. No significant differentially methylated loci were found in the oft-cited MT2 region within the CpG island or across exon 3, even at single CpG positions that previous studies have shown to be frequently altered in autism

Region	Hg38chr3:	bp	CpGs	5mC modification		diff	diff.se	stat	pval
				HFA	NC				
MT2	8,769,033–8769438	405	26	0.156	0.153	0.003	0.022	0.132	0.630
Exon 3	8,768,329–8,767,266	1064	108	0.141	0.148	0.007	0.020	0.401	0.571
**bp—TSS**									
− 860	8,769,047			0.247	0.209	0.038	0.032	1.194	0.232
− 901	8,769,088			0.677	0.660	0.017	0.037	0.462	0.644
− 924	8,769,111			0.517	0.512	0.005	0.037	0.148	0.882
− 934	8,769,121			0.517	0.512	0.005	0.037	0.148	0.882
− 959	8,769,146			0.368	0.381	0.013	0.036	-0.359	0.719
− 982	8,769,169			0.095	0.089	0.006	0.021	0.287	0.774
− 989	8,769,176			0.095	0.089	0.006	0.021	0.286	0.774

In addition, no significant differences in OXTR 5mC modification were found between the sexes (data not shown).

**Fig. 4:**
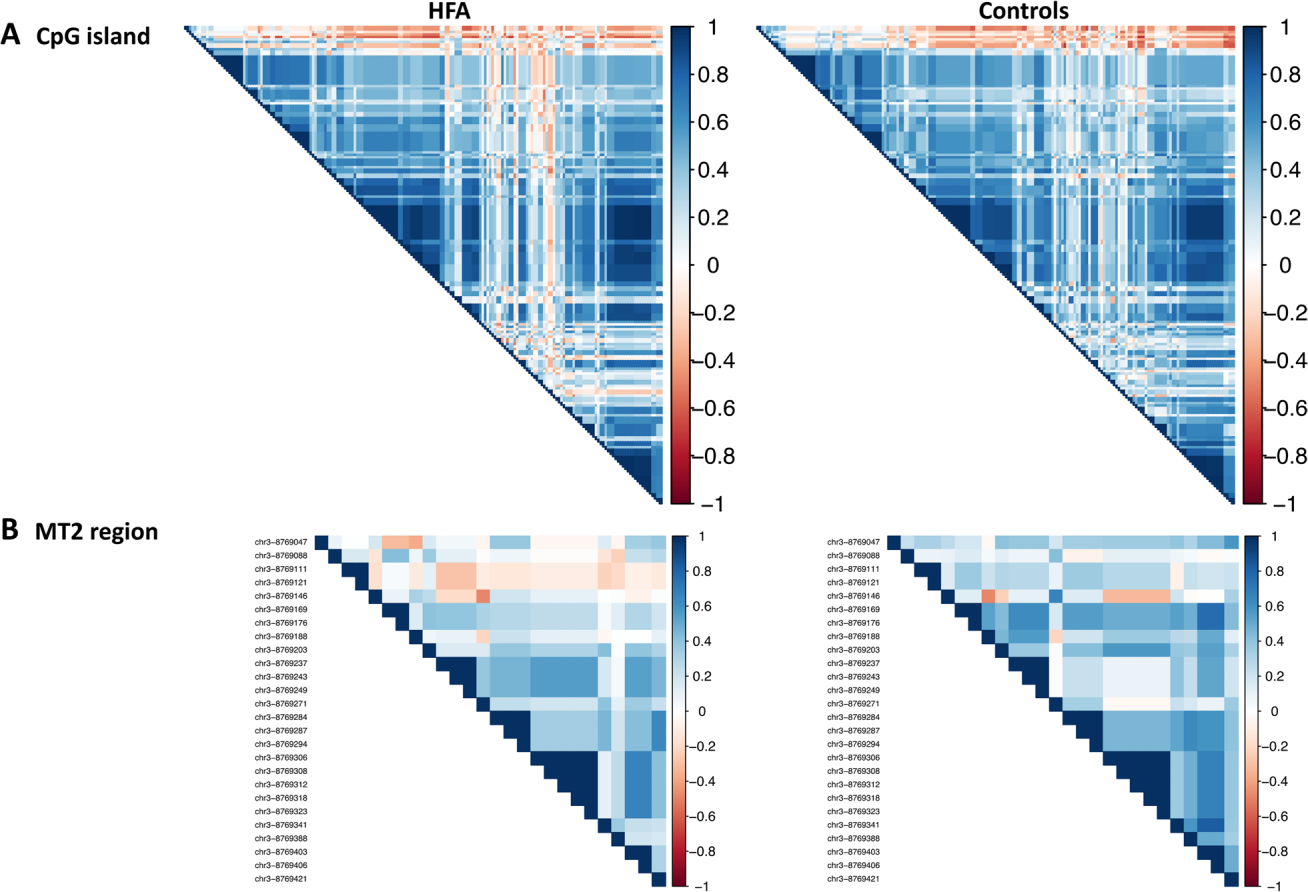
shows the intercorrelation analysis between individual CpG methylation probabilities within the regulatory CpG island (**A**), which contains almost half of all CpGs, and the MT2 region (**B**). CpGs in the 3' portion of MT2, especially chr3-8,769,111 and chr3-8,769,121, show a distinct intercorrelation pattern between groups

As the 5mC modification within the OXTR CpG island has been previously described to be highly intercorrelated [17], we further compared the intercorrelation patterns of the OXTR CpG island and in particular the regulatory relevant MT2 region 5mC modification at included CpG sites between the HFA and NC groups. Figure 4 shows the 5mC modification intercorrelation plots of CpGs within CpG island, respectively, MT2 area of both HFA and NC group. Visually, there is an overall consistent pattern of correlating neighboring CpG mean methylation rates within the CpG island. Focusing on the MT2 region, we see highly intercorrelated neighboring CpGs (8,769,237–8,769,249, 8,769,284–8,769,294 and 8,769,306–9,769,323) almost identically in HFA and NC. Yet, CpGs 8,769,047–8,769,146 visually exhibit a more negative correlation with the other MT2 region CpGs in the HFA group compared to NC. However, differential methylation analysis also revealed no significant differences between the groups at these CpG loci -901 (67.7% ± 13.6 in HFA to 66.0 ± 10.5 in NC), -924 (51.7% ± 12.4 to 51.2 ± 14.1) and -934 (51.7% ± 12.4 to 51.2 ± 14.1). Figure 3B shows the distribution of data for these three CpG positions analogous to 2A as combined box and violin plots comparing groups.

### DNA sequence variation

Group-dependent visualization of the detected SNPs within OXTR showed clustering of the SNP rs918316 (T > C) in the HFA group. The difference in clustering between groups was marginally significant for rs918316 with heterozygosity in a quarter of the HFA subjects, χ^2^(1) = 5.714, p (Fisher exact) = 0.047, φ = − 0.378, corresponding to a Cohen's medium effect.

For the SNP rs53576 within OXTR intron 3 (A > G,T; Hg38, chr3:8,762,685), which has been most frequently described in the context of autism, altered social behavior and also differential OXTR methylation [4], no group-dependent clustering was observed in this study. Similarly, other SNPs within the OXTR sequence previously found to be associated with ASD [3], such as rs7632287, rs237887, rs2268491, rs2254298 and rs237902 did not differ significantly in a group-dependent manner here. Table 4 summarizes the results.

**Table 4:** **A** List of detected SNPs that appeared to cluster toward the HFA group in the IGV visualization. rs918316 frequency was shown to be significantly different between groups. **B** No group differences were found for SNPs previously associated with ASD (and partly with differential OXTR methylation)

	SNP	Position (chr3:)	Alleles	Genotype	Group		Statistic		
A					HFA	NC	Chi^2^ test		
					n SNP (%)		χ^2^	df	p
	**rs918316**	8,756,495	T > C	T|C/C|T	5 (12.5%)	0 (0%)	5.714	1	**0.047**
B									
	**rs7632287**	8,749,760	G > A	A|G/G|A	0	0	-	-	-
	**rs237887**	8,755,356	G > A		14 (35%)	13 (32.5%)	0.418	2	0.811
	Heterozygous			A|G/G|A	9	7			
	Homozygous			A|A	5	6			
	**rs2268491**	8,758,712	C > T	C|T	2 (5%)	0 (0%)	2.105	1	0.487
	**rs2254298**	8,760,542	G > A	G|A	2 (5%)	0 (0%)	2.105	1	0.487
	**rs53576**	8,762,685	A > G		14 (35%)	13 (32.5%)	0.477	2	0.788
	Heterozygous			A|G/G|A	7	8			
	Homozygous			G|G	7	5			
	**rs237902**	8,767,498	G > A		13 (32.5%)	8 (20%)	3.649	2	0.161
	Heterozygous			A|G	12	6			
	Homozygous			A|A	1	2			

## Discussion

The present study investigated sequence variation and methylation of the entire OXTR gene using a targeted nanopore long-read sequencing approach in individuals with HFA compared to NC.

In contrast to several previous studies describing an association of differential OXTR methylation with autism, no group differences in OXTR methylation could be detected within our data. Given the large number of positive results, especially regarding the association of OXTR MT2 region methylation with autism, it was surprising to us that none of the previous results could be reproduced here. In contrast, we showed that 5mC modification of the OXTR gene is remarkably consistent across the entire OXTR gene between groups, especially considering the small group size of 20 subjects with HFA compared to 20 NC. Given this consistency of 5mC modification rate across a sequence of 20,054 base pairs and 412 CpG positions, and our finding that no single CpG position differed statistically significantly between groups, the question arises as to the biological relevance of individual methylation changes at single CpG positions and their relevance to autism and autistic-like behavior as reported previously [6].

Consistent with this, the SNP rs53576, which has been frequently associated with OXTR methylation and phenotypic relevance in autism [6], did not show significant group differences in SNP frequency within the DNA sequence analysis. In addition, other SNPs commonly associated with autism did not show significant group differences in our nanopore dataset. The only SNP that was statistically significantly clustered between groups in HFA, rs918316, has not been reported in any publication in relation to autism or other clinical relevance. It is an intron variant at position chr3:8,756,495 (C instead of T, frequencies in the total population according to ALFA T = 0.917 and C = 0.083). Although the total number of cases is small, the clustering found here in 25% of HFA individuals and none of the NC seems worth reporting in the overall context.

Three distinct CpG methylation sites within the MT2 region of OXTR have previously been associated with ASD, as well as with OXTR expression, and have been shown to be highly correlated with total MT2 methylation (Danoff et al., 2021). Interestingly, the same CpGs -901, -924 and -934 (corresponding to positions chr3-8,769,088, -8,769,111 and -8,769,121 in Fig. 2B), which have been described as important regulators of OXTR expression, appear to be differentially intercorrelated in our analysis in the HFA group compared to the NC group, with a positive correlation to neighboring MT2 CpG sites in NC, but a negative correlation in the HFA group.

Could intercorrelation changes of the OXTR MT2 region be associated with autism? Intercorrelation between adjacent CpGs allows for strong, error-tolerant inheritance of methylation patterns. This enhances the recruitment of demethylating enzymes by unmethylated CpGs in the neighborhood, allowing CpG islands to remain hypomethylated within predominantly hypermethylated entire gene segments [18]. Although we did not find group differences in methylation, we could at least hypothesize a weakening of the hypomethylation robustness of this important regulatory region in individuals with HFA compared to healthy NC. Figure 3B also shows overall partially divergent correlation patterns between CpGs within the OXTR CpG island between HFA and NC. However, the relevance of CpGs -901, -924 and -934 for OXTR expression postulated in preliminary studies is questioned in a recent study. Siecinski et al. analyzed three CpG sites within the MT2 region of OXTR previously associated with ASD and OXTR expression (-901, -924, -934) by targeted pyrosequencing, but found no significant association with plasma oxytocin levels [19].

We can also see that from a methodological point of view, the absolute methylation levels found in ASD individuals vary widely between the present study and previous studies, implying that different sequencing methods were used. Factors such as study design, age or sex distribution of the study cohorts certainly play a role. However, the influence of the accuracy of the sequencing methods should not be neglected, especially since we have carefully controlled for the important factors of age and sex. Could the lack of reproducibility of the previously found methylation differences and sequence variations also be methodological?

In our opinion, the results presented here highlight the importance of discussing the methodological limitations of methods such as the widely used bisulfite sequencing, depending on factors such as the structure of the target of interest, the level of methylation and the size of the cohort. Bisulfite sequencing degrades large amounts of DNA by depyrimidination under the required acidic and thermal conditions, which limits the power if the amount of DNA in the sample is low to begin with [20]. Furthermore, bisulfite sequencing relies on the complete conversion of unmodified cytosine to thymine, which makes up the majority of the total cytosine in the human genome, which significantly reduces the complexity of the sequence and can lead to poor sequencing quality [21]. Preliminary results of a comparative study from our laboratory suggest an advantage of nCATS over direct Sanger bisulfite sequencing for small cohort sizes and larger gene targets. However, the authors noted that bisulfite sequencing is still an overall valid method for DNA methylation analysis [22].

### Limitations

Of course, it must be discussed that the nCATS method has only been used in a few clinical studies and, to our knowledge, not in any autism study, which makes comparability with previous studies difficult. Data on the accuracy of nanopore long-read sequencing, particularly nCATS, in clinical samples are sparse. The efficiency of nCATS has been reported to be highly variable [23]. The overall mean coverage of 29.233 achieved here is at the lower end of the range, but still corresponds to valid sequencing results, at least for confirmatory analysis [24, 25]. Coverage was stable over the entire genomic region and the CpGs within it. Over the MT2 and exon 3 regulatory regions, coverage was consistently > 30. However, the variability in coverage between samples (as shown in Fig. 1A) may have affected the sensitivity of the 5mC estimates, although the statistical model used takes coverage into account [26]. There was no statistically significant association between mean coverage and case–control status. The variability in coverage appears to be partly due to the concentration of the input DNA, which could be considered in future studies. However, in a preliminary study, the influence of coverage on the accuracy of methylation estimation was described as limited [27]. For the detection of relevant methylation differences at individual CpG positions in the percentage range of 10% and above, as applied here, the algorithm we used showed excellent test performance [28]. The percentage differences in OXTR methylation between ASD and NC in the relevant preliminary studies are in part significantly higher, e.g., 31.9% in Elagoz et al. [7].

Furthermore, as in most previous studies on this topic, peripheral blood material was used for the methylation analysis, which limits the significance of evidence in central processes. While the overall average correlation between brain tissue and peripheral blood material was shown to be quite reliable (0.86), individual CpG sites were shown to be poorly correlated between blood and brain tissue (20.8%) at a nominal significance level of p < 0.05 [29]. However, differential methylation of OXTR in ASD has been detected across different tissues, including in brain tissue [10].

It should also be noted that our approach does not provide any information about the actual gene expression. On the other hand, DNA methylation of MT2 in particular has been described as the most reliable indicator of OXTR gene expression (13), which we did not find to be differentially methylated. However, this needs to be demonstrated in future studies, e.g., by RNA sequencing analysis.

Furthermore, the size of the study cohort must be discussed as it may lead to underpowering. Against this background, the newly detected intron variant has to be interpreted with caution, also because statistically there is only a marginally significant group difference in frequency. The inclusion of OXTR polymorphisms in this pilot study was also intended to illustrate the capabilities of nCATS compared to other methods, rather than to claim to provide valid gene association results with this pilot-related small number of cases. These initial results using nCATS need to be replicated in independent cohorts and with larger sample sizes. Ultimately, however, we believe that the consistency of our methylation results between groups (across a total of 412 CpG positions) provides sufficient evidence for the validity of the sample size. In addition, the differential methylation testing algorithm used here is described by the authors as particularly suitable for small numbers of cases, since variance and sequencing depth are statistically accounted for[26]. However, this implies the absolute necessity of an accurate clinical diagnosis of subjects with autism at recruitment. Especially for diagnosis of HFA in adulthood, guidelines (neither the German S2-guideline nor the British NICE-guideline) give no clear recommendation regarding diagnostic tools. The Adult Asperger Assessment used here refers to a prospectively outdated diagnosis but is comparable to the ICD-11 diagnosis of autism spectrum disorder without cognitive delay and language impairment. In general, it should be kept in mind that a specific high-functioning subgroup of individuals with autism has been studied here, and their results may not necessarily be comparable to those of more severely affected individuals. Nevertheless, OXTR sequence variation and differential methylation have also been explicitly reported in studies focusing exclusively on HFA [30, 31].

## Conclusion

In conclusion, using a nanopore long-read sequencing approach, we found no significant sequence variation and differential methylation between individuals with HFA and NC within the entire OXTR gene, including its regulatory units, except for a previously undescribed intron variant. Interestingly, however, all previous findings of autism-related OXTR SNPs and altered 5mC methylation were not reproducible with our method. In our opinion, this might lead to a reconsideration of the relevance of altered methylation at individual OXTR CpG positions in autism research. However, further validation between nanopore and the previously used sequencing methods is required in order to better classify the results. Moreover, given the pilot nature of the study, these results need to be replicated in independent cohorts and with larger sample sizes.

## Methods

### Clinical assessment

The study included 20 adults with a diagnosis of ICD-10: F84.5 (Asperger Syndrome), referred to as the HFA group. Recruitment took place at an outpatient center specializing in the diagnosis of autism spectrum disorders in adults. The study protocol adhered to the Declaration of Helsinki and was prospectively reviewed and approved by the local ethics committee (approval number 3054-2016). All subjects gave written informed consent to participate in the study.

The diagnostic procedure for autism was based on the German guideline for the diagnosis of ASD. A multi-professional team, including at least two experienced clinicians, performed the diagnostic assessment. The diagnostic interview was based on the Cohen Adult Asperger Assessment (AAA), which is based on the results of the Autism Quotient (AQ) and Empathy Quotient (EQ) self-report questionnaires [32].

The German version of the Wechsler Adult Intelligence Scale—IV (WAIS-IV) was used to determine the intelligence quotient.

Patients with HFA between 18 and 65 years of age who fulfilled the diagnostic criteria according to ICD-10: F84.5 and AAA were included.

In addition, 20 NC were recruited and matched for sex, age (± 5 y) and IQ (± 15 points on the WAIS-IQ total scale). Except for the AAA interview, NC underwent the same testing procedure.

Subjects with intelligence < 70 points on the total IQ scale were excluded from the study.

### Molecular analysis

DNA was extracted from EDTA blood collected from participants by peripheral venipuncture. DNA extraction was performed by the Hannover Unified Biobank using the ChemagicStar DNA-Blood1k kit (PerkinElmer chemagen Technology, Baesweiler, Germany) on a Hamilton ChemagicStar (Hamilton Germany Robotics, Graefelfing, Germany).

For sequencing OXTR, we used nanopore Cas9-targeted sequencing (nCATS), an enrichment strategy that utilizes targeted cleavage of DNA with Cas9 to bind adapters for nanopore sequencing [15]. In principle, adaptive sampling methods such as read until are available, but were not considered effective due to the size of the target of interest here. Amplification-based nanopore methods would have resulted in a loss of methylation data and were therefore not suitable.

Sequence-specific "guide" RNAs (crRNA) were designed using the software Geneious (Biomatters, Inc., Boston, MA, USA) and the “Alt-R Custom Cas9 crRNA Design Tool” (IDT®, Coralville, IA, USA) to cover the entire OXTR gene including relevant regulatory units spanning Hg38: chr3: 8,750,381—8,770,434. crRNAs were produced by IDT®. To optimize coverage, a total of four guide RNAs were positioned staggered over the target region, resulting in two OXTR fragments spanning chr3: 8.770.937–8.763.067 and chr3: 8.761.480–8.749.344. A table with the sequences and genomic position of the guides used and their on-target and off-target scores, which provide information on editing performance, can be found in the Additional file 1.

Cas-mediated PCR-free enrichment was performed according to the manufacturer's protocol with minor adjustments. Products not otherwise specified below were purchased from Oxford Nanopore Technologies, Oxford, UK.

To prevent unspecific ligation of adapters in later steps (besides the target of interest), genomic DNA was first dephosphorylated using NEB Quick calf intestinal phosphatase (CIP) dissolved in NEB CutSmart Buffer and incubated using a T100 Thermocyler (BioRad®) at 37 °C for 30 min and 80 °C for 5 min. Cas9 ribonucleoprotein complexes (RNPs), Taq polymerase and desoxy-adenosine triphosphate (dATP) were then added to the dephosphorylated genomic DNA samples. This process activates the Cas9 cleavage at the predetermined sites for ligation, cutting all available DNA ends in a single step incubated at 37 °C for 30 min and 72 °C for 7 min (slight deviation from the manufacturer's protocol). Previously, Cas9 is loaded with the pre-designed crRNA, which forms an RNP complex with the trans-activating CRISPR RNA (tracrRNA) required for catalytic activity, and then incubated at room temperature for 30 min with NEB CutSmart buffer and nuclease-free water (NFW) to form the Cas9 RNPs. Deviating from the original protocol, we used an amount of 0.6 µl Cas9 instead of 0.4 µl for the preparation of a master mix for five samples to optimize subsequent sequencing coverage. Next, native barcodes (Native Barcoding Expansion 1–12, ONT sequencing, location) were ligated to the ends generated by Cas9 cleavage. A unique barcode was selected for each sample and five samples/barcodes were used within one flow cell. Native barcode ligation mixture containing barcode, Blunt/TA Ligase Master Mix and NFW was added to the cleaved and dA-tailed genomic DNA samples and incubated for 30 min at room temperature and then purified using AMPure XP beads (Beckman Coulter, Brea, CA, USA). Based on our experience, we used 5 µl instead of 3 µl barcode per sample (respectively, combined with 3 µl instead of 5 µl nuclease-free water) to further optimize coverage. After clean-up of the reaction (by means of AMPure XP beads), samples were combined. AMII adapters from the Ligation Sequencing Kit were then ligated to the ends generated by Cas9 cleavage using NEBNext Quick T4 DNA Ligase dissolved in Ligation Buffer (LNB) and then resuspended in AMPure XP beads for purification to remove excess unligated adapters and other short DNA fragments. After control of the genomic DNA concentration, MinION Flow Cells (R9.4.1) were prepared according to the manufacturer's protocol and loaded with the barcoded OXTR DNA library dissolved in sequencing buffer using the supplied loading beads. The flow cell was then processed using the MinION nanopore sequencer and Nanopore MinKnow Sequencing software.

### Data management and statistics

Nanopore raw reads (fast5 format) were basecalled using Guppy Basecalling Software Version 6.1.2 (Oxford Nanopore Technologies, Oxford, UK) and aligned to the reference genome Hg38 using minimap2 [33].

Sequence coverage within the genomic region under study was obtained using the getCoverage function of the bssseq package for R [34] and visualized for distribution per sample and across genomic positions.

Nanopolish was used for cytosine methylation calling [35]. Cytosine modification probability was obtained and visualized for the whole and regions of interest within OXTR gene using NanoMethViz for R [36]. BSSSeq-type objects obtained via the NanoMethViz to BSSSeq interface (methy_to_bssseq) were processed for statistical testing of differentially methylated loci using DSS for R. To determine differential methylation, statistical tests were performed at each CpG site to determine differentially methylated loci (DML) and differentially methylated regions (DMR). According to the authors of the DSS package used here, most existing methods for DM analysis are based on ad hoc tests that, for example, ignore biological variation when using Fisher's exact and sequencing depth when using t tests for estimated methylation values. The DM detection implemented in DSS is based on a Wald test for beta-binomial distributions. The test statistic depends on the biological variation (characterized by the dispersion parameter) and the sequencing depth. An important part of the algorithm is the estimation of the dispersion parameter, which is achieved by an estimator based on a Bayesian hierarchical model. [26, 37, 38]. Therefore, the method is described as being particularly suitable for small sample sizes. The underlying package R codes are available from https://github.com/haowulab/DSS.

In summary, the differential methylation test was performed in the following steps

1. Read the library (object of the BSSSeq class obtained by the NanoMethViz methy_to_bssseq function).
2. Perform statistical tests on the DML by calling the DML test function
  - Estimate the average methylation level across all CpG sites
  - estimate the dispersion at each CpG site, and
  - perform the Wald test
    (For whole genome datasets, statistical smoothing of the data is useful, which was omitted in this case due to the size of the target region (flag smoothing = FALSE)).
3. Based on the test results, DMLs have been called using the callDML function. By default, the test is based on the null hypothesis that the difference in methylation levels is 0. However, the user can specify a threshold for the difference. We examined our data for differences of > 10% between the groups in the estimated mean methylation (flags p.threshold = 0.05, delta = 0.1). Thus, the function calculates the probability that the difference in mean methylation values is greater than delta (here 10%) at a 5% significance level.
4. DMR detection using the callDMR function is also based on the DML test results.

The default requirements for DMRs are a minimum length of 50 bps, a minimum number of CpGs of 3, and a minimum percentage of significantly group-different CpGs within the DMR of 50%. We have again used a group difference in mean methylation of at least 10% as a threshold value (flags p.threshold = 0.05, delta = 0.1).

The same procedure was used to compare methylation data between the sexes.

Methylation data were further visualized in group comparisons using the NanoMethViz plot_region function. Gene annotations were obtained from the UCSC Genome Browser on Human (GRCh38/hg38) [39]. The customized UCSC tracks used to plot our data can be accessed from https://genome.ucsc.edu/s/JWieting/OXTR.

Single-nucleotide variants (SNVs) were called by longshot [40]. The output individual vcf files per sample were merged using bcftools [41] and visualized using the Integrative Genomics Viewer (IGV, Reference Genome GRCH38/hg38) [42], with detected SNVs highlighted by color depending on zygosity. HFA and NC were annotated, output by group, and visually inspected for group-dependent clustering of SNPs. We also looked explicitly for group differences in SNPs already known to be associated with autism. We compared frequency differences between the groups using Chi-squared tests.

The data processing and analysis codes used here are publicly available at https://github.com/wietingj/nanoporeCATS_OXTR.

### Supplementary Information

**Additional file 1. S1.** Guide RNAs used for Cas-mediated PCR-free enrichment of the OXTR sequence analyzed via nanopore sequencing. On-target scores indicate the predicted editing performance at the intended target site (high value is better). High off-target scores indicate lower off-target risk (0-100).

## Data Availability

The data on which the results of this study are based can be made available upon individual request, subject to compliance with the European Union's General Data Protection Regulation.

## References

[1] Yang S, Dong X, Guo X, Han Y, Song H, Gao L, et al. Serum oxytocin levels and an oxytocin receptor gene polymorphism (rs2254298) indicate social deficits in children and adolescents with autism spectrum disorders. Front Neurosci. 2017;11(APR) 10.3389/fnins.2017.00221 PMC5399030 28484366

[2] Parker KJ, Garner JP, Libove RA, Hyde SA, Hornbeak KB, Carson DS (2014). Plasma oxytocin concentrations and OXTR polymorphisms predict social impairments in children with and without autism spectrum disorder. Proc Natl Acad Sci U S A.

[3] LoParo D, Waldman ID (2014). The oxytocin receptor gene (OXTR) is associated with autism spectrum disorder: a meta-analysis. Mol Psychiatry..

[4] Rijlaarsdam J, van Ijzendoorn MH, Verhulst FC, Jaddoe VWV, Felix JF, Tiemeier H (2017). Prenatal stress exposure, oxytocin receptor gene (OXTR) methylation, and child autistic traits: the moderating role of OXTR rs53576 genotype. Autism Res.

[5] Deaton AM, Bird A (2011). CpG islands and the regulation of transcription. Genes Dev.

[6] Moerkerke M, Bonte M-L, Daniels N, Chubar V, Alaerts K, Steyaert J (2021). Oxytocin receptor gene (OXTR) DNA methylation is associated with autism and related social traits—a systematic review. Res Autism Spectr Disord..

[7] ElagozYuksel M, Yuceturk B, FarukKaratas O, Ozen M, Dogangun B (2016). The altered promoter methylation of oxytocin receptor gene in autism. J Neurogenet.

[8] Siu MT, Goodman SJ, Yellan I, Butcher DT, Jangjoo M, Grafodatskaya D (2021). DNA methylation of the oxytocin receptor across neurodevelopmental disorders. J Autism Dev Disord.

[9] Andari E, Nishitani S, Kaundinya G, Caceres GA, Morrier MJ, Ousley O (2020). Epigenetic modification of the oxytocin receptor gene: implications for autism symptom severity and brain functional connectivity. Neuropsychopharmacology.

[10] Gregory SG, Connelly JJ, Towers AJ, Johnson J, Biscocho D, Markunas CA, et al. Genomic and epigenetic evidence for oxytocin receptor deficiency in autism. BMC Med. 2009;7 10.1186/1741-7015-7-62 PMC2774338 19845972

[11] Kusui C, Kimura T, Ogita K, Nakamura H, Matsumura Y, Koyama M (2001). DNA methylation of the human oxytocin receptor gene promoter regulates tissue-specific gene suppression. Biochem Biophys Res Commun.

[12] Perkeybile AM, Carter CS, Wroblewski KL, Puglia MH, Kenkel WM, Lillard TS (2019). Early nurture epigenetically tunes the oxytocin receptor. Psychoneuroendocrinology.

[13] Danoff JS, Wroblewski KL, Graves AJ, Quinn GC, Perkeybile AM, Kenkel WM (2021). Genetic, epigenetic, and environmental factors controlling oxytocin receptor gene expression. Clin Epigenetics.

[14] Ziegler C, Dannlowski U, Bräuer D, Stevens S, Laeger I, Wittmann H (2015). Oxytocin receptor gene methylation: converging multilevel evidence for a role in social anxiety. Neuropsychopharmacology.

[15] Gilpatrick T, Lee I, Graham JE, Raimondeau E, Bowen R, Heron A (2020). Targeted nanopore sequencing with Cas9-guided adaptor ligation. Nat Biotechnol.

[16] de Giambattista C, Ventura P, Trerotoli P, Margari F, Margari L (2021). Sex differences in autism spectrum disorder: focus on high functioning children and adolescents. Front Psychiatry.

[17] Müller S, Sicorello M, Moser D, Frach L, Limberg A, Gumpp AM (2022). The DNA methylation landscape of the human oxytocin receptor gene (OXTR): Recommendations for future research. BioRxiv..

[18] Haerter JO, Lövkvist C, Dodd IB, Sneppen K (2014). Collaboration between CpG sites is needed for stable somatic inheritance of DNA methylation states. Nucleic Acids Res.

[19] Siecinski SK, Giamberardino SN, Spanos M, Hauser AC, Gibson JR, Chandrasekhar T (2023). Genetic and epigenetic signatures associated with plasma oxytocin levels in children and adolescents with autism spectrum disorder. Autism Res.

[20] Tanaka K, Okamoto A (2007). Degradation of DNA by bisulfite treatment. Bioorg Med Chem Lett.

[21] Olova N, Krueger F, Andrews S, Oxley D, Berrens RV, Branco MR (2018). Comparison of whole-genome bisulfite sequencing library preparation strategies identifies sources of biases affecting DNA methylation data. Genome Biol.

[22] Gombert S, Jahn K, Pathak H, Burkert A, Schmidt G, Wiehlmann L (2023). Comparison of methylation estimates obtained via MinION nanopore sequencing and sanger bisulfite sequencing in the TRPA1 promoter region. BMC Med Genomics.

[23] Skowronek D, Pilz RA, Bonde L, Schamuhn OJ, Feldmann JL, Hoffjan S (2022). Cas9-mediated nanopore sequencing enables precise characterization of structural variants in CCM genes. Int J Mol Sci..

[24] Radhakrishnan GV, Cook NM, Bueno-Sancho V, Lewis CM, Persoons A, Mitiku AD (2019). MARPLE, a point-of-care, strain-level disease diagnostics and surveillance tool for complex fungal pathogens. BMC Biol.

[25] De La Cerda GY, Landis JB, Eifler E, Hernandez AI, Li FW, Zhang J, et al. Balancing read length and sequencing depth: optimizing nanopore long‐read sequencing for monocots with an emphasis on the Liliales. Appl Plant Sci. 2023;11(3) 10.1002/aps3.11524 PMC10278932 37342170

[26] Feng H, Wu H (2019). Differential methylation analysis for bisulfite sequencing using DSS. Quant Biol (Beijing, China).

[27] Wing-Sze Yuen Z, Srivastava A, Daniel R, McNevin D, Jack C, Eyras E (2022). Systematic benchmarking of tools for CpG methylation detection from nanopore sequencing. Nat Commun.

[28] Zhang Y, Baheti S, Sun Z (2018). Statistical method evaluation for differentially methylated CpGs in base resolution next-generation DNA sequencing data. Brief Bioinform..

[29] Braun PR, Han S, Hing B, Nagahama Y, Gaul LN, Heinzman JT (2019). Genome-wide DNA methylation comparison between live human brain and peripheral tissues within individuals. Transl Psychiatry.

[30] Nyffeler J, Walitza S, Bobrowski E, Gundelfinger R, Grünblatt E. Association study in siblings and case-controls of serotonin- and oxytocin-related genes with high functioning autism. J Mol Psychiatry. 2014;2(1) 10.1186/2049-9256-2-1 PMC4223888 25408912

[31] Wermter AK, Kamp-Becker I, Hesse P, Schulte-Körne G, Strauch K, Remschmidt H (2010). Evidence for the involvement of genetic variation in the oxytocin receptor gene (OXTR) in the etiology of autistic disorders on high-functioning level. Am J Med Genet B Neuropsychiatr Genet..

[32] Baron-Cohen S, Wheelwright S, Robinson J, Woodbury-Smith M (2005). The adult asperger assessment (AAA): A Diagnostic method. J Autism Dev Disord..

[33] Li H (2018). Minimap2: pairwise alignment for nucleotide sequences. Bioinformatics.

[34] Hansen KD, Langmead B, Irizarry RA (2012). BSmooth: from whole genome bisulfite sequencing reads to differentially methylated regions. Genome Biol.

[35] Loman NJ, Quick J, Simpson JT (2015). A complete bacterial genome assembled de novo using only nanopore sequencing data. Nat Methods.

[36] Su S, Gouil Q, Blewitt ME, Cook D, Hickey PF, Ritchie ME. NanoMethViz: An R/Bioconductor package for visualizing long-read methylation data. PLoS Comput Biol. 2021;17(10) 10.1371/journal.pcbi.1009524 PMC8568149 34695109

[37] Feng H, Conneely KN, Wu H (2014). A Bayesian hierarchical model to detect differentially methylated loci from single nucleotide resolution sequencing data. Nucleic Acids Res.

[38] Wu H, Xu T, Feng H, Chen L, Li B, Yao B, et al. Detection of differentially methylated regions from whole-genome bisulfite sequencing data without replicates. Nucleic Acids Res. 2015;43(21) 10.1093/nar/gkv715 PMC4666378 26184873

[39] Kent WJ, Sugnet CW, Furey TS, Roskin KM, Pringle TH, Zahler AM (2002). The human genome browser at UCSC. Genome Res.

[40] Edge P, Bansal V (2019). Longshot enables accurate variant calling in diploid genomes from single-molecule long read sequencing. Nat Commun.

[41] Danecek P, Bonfield JK, Liddle J, Marshall J, Ohan V, Pollard MO, et al. Twelve years of SAMtools and BCFtools. Gigascience. 2021;10(2) 10.1093/gigascience/giab008 PMC7931819 33590861

[42] Thorvaldsdóttir H, Robinson JT, Mesirov JP (2013). Integrative Genomics Viewer (IGV): high-performance genomics data visualization and exploration. Brief Bioinform..

