# Supplementary material for: A targeted long-read sequencing approach questions the association of OXTR methylation with high-functioning autism
Source: Clin Epigenetics. 2023 Dec 20;15:195. doi: 10.1186/s13148-023-01616-4 (PMC10734107; doi:10.1186/s13148-023-01616-4)
Supplement: Supplementary file 1 — Additional file 1. S1. Guide RNAs used for Cas-mediated PCR-free enrichment of the OXTR sequence analyzed via nanopore sequencing. On-target scores indicate the predicted editing performance at the intended target site (high value is better). High off-target scores indicate lower off-target risk (0-100). [file 13148_2023_1616_MOESM1_ESM.docx]

**SUPPLEMENTARY MATERIAL**

SUPPLEMENTARY MATERIAL S1

| Lab-ID | Sequence | PAM | GRCH38 genomic position | On-target score | Off-target  score |
| --- | --- | --- | --- | --- | --- |
| 67 | GGGCTACGCGAGGACCACAC | AGG | Chr. 3: 8770937 to 8770918 | 67 | 83 |
| 68 | CCCAAGAGTCTTATAACTCC | AGG | Chr. 3: 8749344 to 8749363 | 82 | 65 |
| 77 | TGGACTTGGGCTTAACAAGT | AGG | Chr. 3: 8763067 to 8763086 | 76 | 68 |
| 78 | GATGATCTACACCTTCGACT | TGG | Chr. 3: 8761480 to 8761461 | 82 | 87 |

PAM: Protospacer Adjacent Motif.

**S1** Guide RNAs used for Cas-mediated PCR-free enrichment of the *OXTR* sequence analyzed via nanopore sequencing. On-target scores indicate the predicted editing performance at the intended target site (high value is better). High off-target scores indicate lower off-target risk (0 – 100).
